# Immune-Related LncRNAs as Prognostic Factors for Pediatric Rhabdoid Tumor of the Kidney

**DOI:** 10.1155/2022/4752184

**Published:** 2022-06-15

**Authors:** Ye Hong, Yi Que, Yang Hu, Bo-yun Shi, Jia Zhu, Juan Wang, Jun-ting Huang, Fei-fei Sun, Lian Zhang, Xin-ke Zhou, Su-ying Lu, Yi-zhuo Zhang

**Affiliations:** ^1^State Key Laboratory of Oncology in South China, Collaborative Innovation Center for Cancer Medicine, Sun Yat-Sen University Cancer Center, Guangzhou 510060, China; ^2^Department of Pediatric Oncology, Sun Yat-Sen University Cancer Center, Guangzhou 510060, China; ^3^Department of Pediatric, The Fifth Affiliated Hospital, Guangzhou Medical University, 621 GangWan Road, Guangzhou, Guang dong 510700, China

## Abstract

**Background:**

Immune-related long noncoding RNAs (IrlncRNAs) are recognized as important prognostic factors in a variety of cancers, but thus far, their prognostic value in pediatric rhabdoid tumor of the kidney (pRTK) has not been reported. Here, we clarified the associations between IrlncRNAs and overall survival (OS) of pRTK patients and constructed a model to predict their prognosis.

**Methods:**

We accessed RNA sequencing data and corresponding clinical data of pRTK from the Therapeutically Applicable Research to Generate Effective Treatments (TARGET) database. An expression profile of immune-related genes (Irgenes) and lncRNAs of pRTK was extracted from the RNA sequencing data. IrlncRNAs were defined by co-expression analysis of lncRNAs and Irgenes. The limma R package was used to identify differential expression IrlncRNAs. Univariate and multivariate Cox regression analyses were conducted to build a prognostic IrlncRNAs model. The performance of this prognostic model was validated by multimethods, like ROC curve analysis.

**Results:**

A total of 1097 IrlncRNAs were defined. Univariate Cox regression analysis identified 7 IrlncRNAs (AC004791.2, AP003068.23, RP11-54O7.14, RP11-680F8.1, TBC1D3P1-DHX40P1, TUNAR, and XXbac-BPG308K3.5) and were significantly associated with OS. Multivariate regression analysis constructed the best prognostic model based on the expression of AC004791.2, AP003068.23, RP11-54O7.14, TBC1D3P1-DHX40P1, and TUNAR. According to the prognostic model, a risk score of each patient was calculated, and patients were divided into high-risk and low-risk groups accordingly. The survival time of low-risk patients was significantly better than high-risk patients (*p* < 0.001). Univariate (hazard ratio 1.098, 95% confidence interval 1.048–1.149, *p* value <0.001) and multivariate (hazard ratio 1.095, 95% confidence interval 1.043–1.150, *p* value <0.001) analyses confirmed that the prognostic model was reliable and independent in prediction of OS. Time-dependent ROC analysis showed that 1-year survival AUC of prognostic model, stage, age, and sex was 0.824, 0.673, 0.531, and 0.495, respectively, which suggested that the prognostic model was the best predictor of survival in pRTK patients.

**Conclusions:**

The prognostic model based on 5 IrlncRNAs was robust and could better predict the survival of pRTK than other clinical factors. Additionally, the mechanism of regulation and action of prognosis-associated lncRNAs could provide new avenues for basic research to explore the mechanism of tumor initiation and development in order to prevent and treat pRTK.

## 1. Introduction

Rhabdoid tumor of the kidney (RTK) is a very rare cancer with a dismal prognosis [1]. Infants and young children are at high risk of RTK, which accounts for 1.3%-2% of kidney tumors in this age group [2–4]. The mainstay treatments for RTK include surgery, chemotherapy, and radiotherapy [5], but even with comprehensive treatments, the prognosis remains extremely poor with 5-year overall survival (OS) no more than 20%-25% [6, 7]. At present, judging the prognosis of RTK is mainly dependent on clinical factors like age and stage [8, 9]. There is a lack of individualized molecular predictors of prognosis in pRTK.

Long noncoding RNAs (lncRNAs) are nonprotein-coding transcripts with >200 nucleotides [10]. LncRNAs account for about 80% of the human transcriptome, which interact with DNA, RNA, and protein to exert a powerful regulatory function, such as epigenetic modification, transcription control, and posttranscriptional modification [11–13]. LncRNAs are involved in essential biological processes within cells, including cell growth, cell differentiation, cell invasion, and cell cycle control [12, 14, 15]. Dysregulation of lncRNA is associated with several diseases [12, 13]. Studies suggest that lncRNAs are deregulated in various cancers and played an important role in occurrence, development, and metastasis [14, 16–20]. Besides, multiple studies have demonstrated that lncRNAs perform a crucial function in T cell and NK cell regulation in malignancies, like hepatocellular cancers and lung cancers [13, 15]. The potential mechanisms of action of lncRNAs in malignancies include chromatin remodeling induction, transcription interference, alternative splicing, production of endo-siRNAs or miRNAs called “miRNA sponges,” and altering the localization or activity of proteins [13]. Based on these mechanisms, the level and function of proteins and their related-signaling pathways, such as phosphatidylinositol 3-kinase (PI3K/Akt) pathway, NF-KB pathway, and tumor necrosis factor (TNF) pathway, may be dysregulated and thus result in the initiation, progression, and abnormal immune infiltration of cancer [12–15]. LncRNAs have been shown to be promising diagnostic biomarkers and therapeutic targets in different cancers [21, 22]. In addition, several studies have also explored and verified the prognostic value of IrlncRNAs in different tumors such as glioma, breast cancer, pancreatic cancer, and others [11, 23–26]. Reported studies have demonstrated that IrlncRNAs are associated with tumor immune cell infiltration which could affect development and metastasis of the tumor and the response to treatment [13].

RTK exhibits an immune-inflamed phenotype, which is characterized by the activation of the immune system, increase of cytotoxic cell infiltration and PD-L1 expression, and augmentation of antigen presentation. These phenotypic features indicate that immune-associated elements might play important roles in RTK [27]. There are no reports concerning the role of IrlncRNAs in pRTK. Thus, we aimed to investigate the association of IrlncRNAs and OS in pRTK and provide a rational basis for clinicians to judge the prognosis of individual patients.

## 2. Materials and Methods

### 2.1. Downloading and Processing Data

RNA sequencing data and corresponding clinical data of pRTK and normal kidney samples were downloaded from the Therapeutically Applicable Research to Generate Effective Treatments (TARGET, https://ocg.cancer.gov/programs/target) database. The Perl programming language was utilized to process the RNA sequencing data and extract the lncRNA data. All data were analyzed by using the R3.6.3 software (https://www.r-project.org/) [28]. A list of immune-related genes (Irgenes) was downloaded from the gene list resources in Immunology Database and Analysis Portal (ImmPort, https://www.immport.org/) [29], and a list of transcription factors (TF) was downloaded from the human transcription factors database (http://humantfs.ccbr.utoronto.ca/download.php).

### 2.2. Identification of Irgenes and IrlncRNAs

R3.6.3 software was utilized to extract expression data of Irgenes from the RNA sequencing data. IrlncRNAs were defined through the co-expression analysis of lncRNAs and Irgenes. Univariate Cox regression analysis was conducted to screen the IrlncRNAs that were significantly associated with OS.

### 2.3. Construction of a Prognostic Model Based on IrlncRNAs

Multivariate Cox analysis was used to identify independent prognosis-associated IrlncRNAs to establish best prognostic model. The risk score of each patient was calculated by using the following formula: risk score = exp1∗coef1 + exp2∗coef2 + ⋯⋯+expn∗coefn, where exp is the expression level of prognostic IrlncRNAs and coef is the regression coefficient of multivariate analysis. The median value of the risk score was then used as the cut-off value, and patients were divided into high-risk and low-risk groups accordingly.

### 2.4. Gene Set Enrichment Analysis and Immune Cell Abundance Identifier Analysis

Gene set enrichment analysis (GSEA) was carried out to identify different functional phenotypes of the high-risk and low-risk groups. Perl was used for extracting mRNA expression data from RNA sequencing data. GSEA was conducted on the mRNA expression profiles of high-risk and low-risk groups. The enriched gene sets within absolute value of normalized enrichment score >1, a nominal *p* < 0.05, and FDR<0.25 were defined meaningfully.

The Immune Cell Abundance Identifier tool (ImmuCellAI, http://bioinfo.life.hust.edu.cn/ImmuCellAI/) was used to infer the relative proportion of 24 types of immune cells in high-risk and low-risk patients.

### 2.5. Evaluation of Drug Sensitivity

R package pRRophetic [30] was utilized to evaluate the sensitivity to common drugs in the high-risk and low-risk groups' pRTK patients. The evaluation index of drug sensitivity was IC50.

### 2.6. Construction of TF Regulator Network

R3.6.3 software was utilized to extract the TF expression profile from the RNA sequencing data. Differential expressed TFs were identified by the limma R package. Prognostic IrlncRNAs-TF pairs were screened out by co-expression analysis of IrlncRNAs and TFs. The Cytoscape software was utilized to visualize the TFs regulatory network.

## 3. Results

### 3.1. Downloading and Processing Data

**Figure 1:**
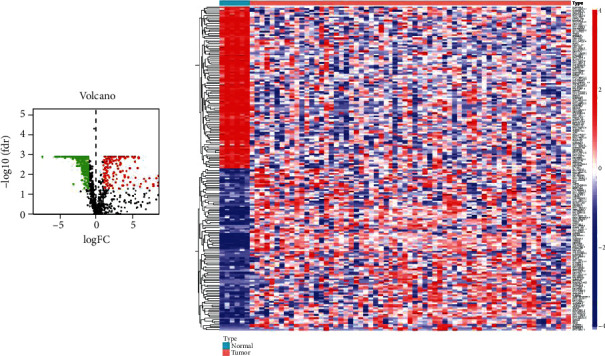
Differentially expressed IrlncRNAs in RTK. (a) Volcano plot of differentially expressed IrlncRNAs in RTK. Colored dots indicate differentially expressed IrlncRNAs and black dots indicate nondifferentially expressed IrlncRNAs. The red dots represent the upregulated gene expression in RTK and the green dots the downregulated. (b) Heat map of differentially expressed IrlncRNAs in RTK. The color from blue to red indicates the gene expression level from low to high. Abbreviations: RTK: rhabdoid tumor of the kidney; IrlncRNAs: immune-related long noncoding RNAs.

First, we download the RNA sequencing data of 65 RTK tissues and 6 normal kidney tissues, containing 50353 transcripts. Second, 19056 transcripts of mRNA and 12053 transcripts of lncRNA were extracted by using Perl. Additionally, clinical data from 65 RTK samples were downloaded. Eight patients without survival data were excluded from this study. The Irgenes' list including 2483 genes was downloaded from the ImmPort database, and the expression data of Irgenes was extracted from RNA sequencing data by R3.6.3 software. Through co-expression analysis of Irgenes and lncRNAs, 1097 IrlncRNAs (correlation coefficient >0.4 and a *p* < 0.001) were identified. Three hundred and ninety-one differentially expressed IrlncRNAs were found between normal and RTK samples through limma R package analysis. The differentially expressed IrlncRNAs were defined as | log FC| ≥1 and FDR<0.05 (Figure 1). Total number of 1595 TFs were downloaded from the human transcription factors database, and 461 differentially expressed TFs in normal and RTK samples were identified by limma R package analysis (|log FC| ≥1 and FDR<0.05).

### 3.2. Identification of Prognostic IrlncRNAs and Construction of a Prognostic Model

**Figure 2:**
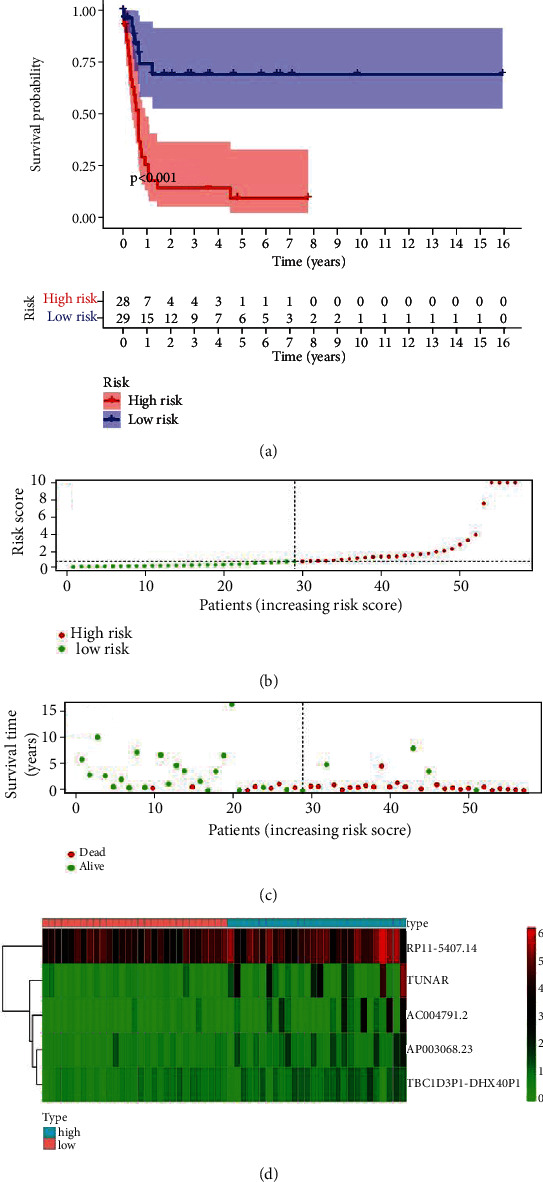
Prognostic analysis of the TARGET-RTK cohort. (a) Kaplan-Meier survival analysis showed that OS of the low-risk group was better than the high-risk group. (b) Dot plot of the risk score. Horizontal and vertical axes represent the RTK patients and corresponding risk score, respectively. Red and green dots represent the high-risk and low-risk patients, respectively. According to risk score, patients were ranked in ascending order on the horizontal axis. (c) Dot plot of OS. Horizontal and vertical axes represented RTK patients and corresponding survival time, respectively. Red and green dots represent dead and alive RTK patients, respectively. According to risk score, patients were ranked in ascending order on the horizontal axis. (d) Heat map of the expression levels of the 5 prognosis-associated IrlncRNAs in high-risk and low-risk patients. Vertical and horizontal axes represent RTK patients and corresponding 5 gene expression levels. Abbreviations: TARGET: Therapeutically Applicable Research to Generate Effective Treatments; RTK: rhabdoid tumor of the kidney; OS: overall survival; IrlncRNAs: immune-related long noncoding RNAs.

**Figure 3:**
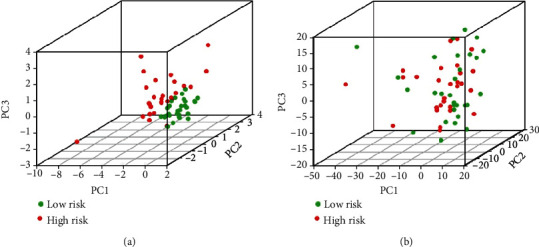
PCA of low-risk and high-risk groups. (a) PCA of low-risk and high-risk groups based on the expression level of prognosis-associated IrlncRNAs. (b) PCA of low-risk and high-risk groups based on the expression level of all IrlncRNAs. Abbreviations: PCA: principal components analysis; IrlncRNAs: immune-related long noncoding RNAs.

Seven IrlncRNAs (AC004791.2, AP003068.23, RP11-54O7.14, RP11-680F8.1, TBC1D3P1-DHX40P1, TUNAR, and XXbac-BPG308K3.5) that were clearly associated with OS (*p* < 0.01) were identified by univariate Cox analysis (Table 1). Based on these results, multivariate Cox analysis was carried out, and 5 IrlncRNAs (AC004791.2, AP003068.23, RP11-54O7.14, TBC1D3P1-DHX40P1, and TUNAR) were identified to establish the best prognostic model (Table 2). Risk score of each RTK patient was calculated based on the expression level of 5 IrlncRNAs as following formula: Risk score = 0.14∗AC004791.2 + 0.49∗AP003068.23 + 0.04∗RP11 − 54O7.14 + 1.11∗TBC1D3P1 − DHX40P1 + 0.06∗TUNAR. According to the median value of the risk score, RTK patients were divided into high-risk and low-risk groups. Kaplan-Meier survival analysis demonstrated that the OS of the low-risk group was significantly better than the high-risk group (*p* < 0.001) (Figure 2). Principal component analysis (PCA) showed a specific distribution pattern of the high-risk and low-risk groups based on the prognosis-associated IrlncRNAs (Figure 3).

### 3.3. Evaluation of the IrlncRNAs Model as an Independent Prognostic Factor

**Figure 4:**
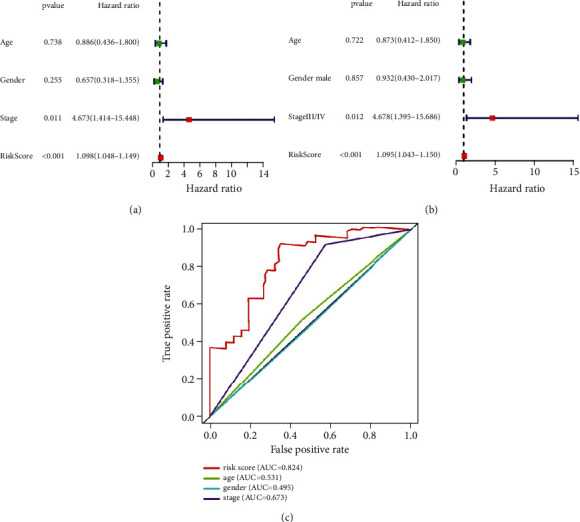
Performance evaluation of risk score. (a) Univariate Cox analysis explored the association of risk score, age, stage, gender, and overall survival in the TARGET cohort. (b) Multivariate Cox analysis explored the association of risk score, age, stage, gender, and overall survival in the TARGET cohort. (c) Time-dependent ROC analysis evaluated the 1-year survival predictive performance of risk score, gender, stage, and age.

Univariate and multivariate analyses were conducted to clarify whether the prognostic model was an independent prognostic factor for OS in RTK patients. Univariate analysis showed risk score (*p* < 0.001) and stage (*p* = 0.011) were significantly associated with OS and multivariate analysis verified both risk score (*p* < 0.001) and stage (*p* = 0.012) were independent prognostic factors for OS. Time-dependent ROC analysis indicated that compared with age (AUC = 0.531), gender (AUC = 0.495), and stage (AUC = 0.673), the risk score (AUC = 0.824) was the best predictor of 1-year survival of RTK patients (Figure 4).

### 3.4. Gene Set Enrichment Analysis and Immune Cell Abundance Identifier Analysis

**Figure 5:**
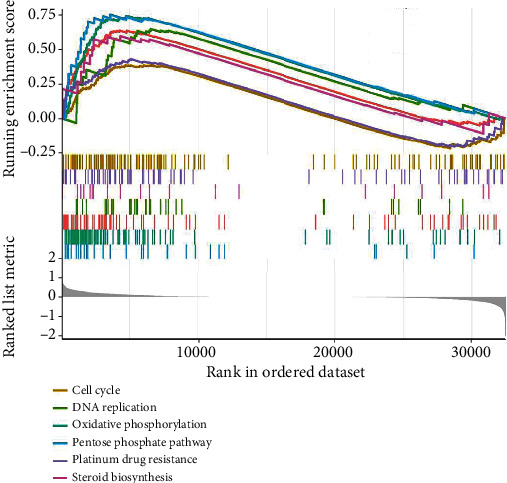
Gene set enrichment analysis (GSEA) indicating cell cycle, DNA replication, pentose phosphate pathway, platinum drug resistance, and steroid biosynthesis was significantly enriched in the high-risk group.

**Figure 6:**
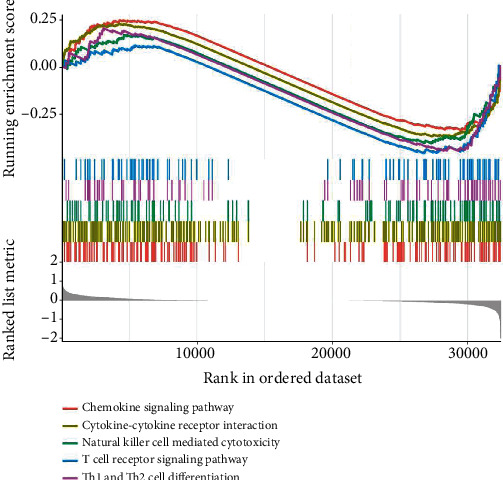
Gene set enrichment analysis (GSEA) indicating cytokine-cytokine receptor interaction, natural killer cell-mediated cytotoxicity, Th1 and Th2 cell differentiation, and T cell receptor signaling pathway was significantly enriched in the low-risk group.

Gene set enrichment analysis (GSEA) was carried out in high-risk and low-risk patients. The results showed that cell cycle, DNA replication, pentose phosphate pathway, platinum drug resistance, and steroid biosynthesis were significantly enriched in the high-risk group (Figure 5), and cytokine-cytokine receptor interaction, natural killer cell-mediated cytotoxicity, Th1 and Th2 cell differentiation, and T cell receptor signaling pathway were significantly enriched in the low-risk group (Figure 6).

**Figure 7:**
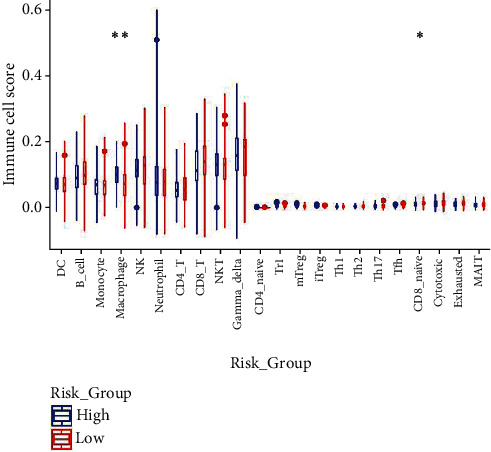
Immune Cell Abundance Identifier analysis indicated that the proportion of macrophages in the high-risk group was higher than in the low-risk group, whereas the distribution of proportion of CD8_naive in the two groups was the reverse.

Immune Cell Abundance Identifier analysis was conducted to infer the relative proportion of 24 types of immune cells of high-risk and low-risk patients. The results revealed that the proportion of macrophages in the high-risk group was higher than in low-risk group, whereas the distribution of proportion of CD8_naive in two groups was the reverse (Figure 7).

### 3.5. Analysis of Drug Sensitivity

**Figure 8:**
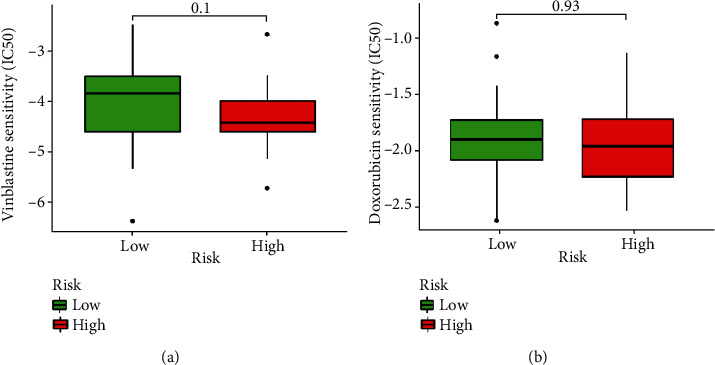
Drug sensitivity analysis of vinblastine (a) and doxorubicin (b) in the low-risk and high-risk groups.

We used R package pRRophetic to evaluate the sensitivity (IC50) of the high-risk and low-risk groups to common drugs that used to treat pRTK. Results suggested that IC50s of vinblastine and doxorubicin were not significantly different between high-risk and low-risk groups (Figure 8).

### 3.6. Construction of the TF Regulatory Network

**Figure 9:**
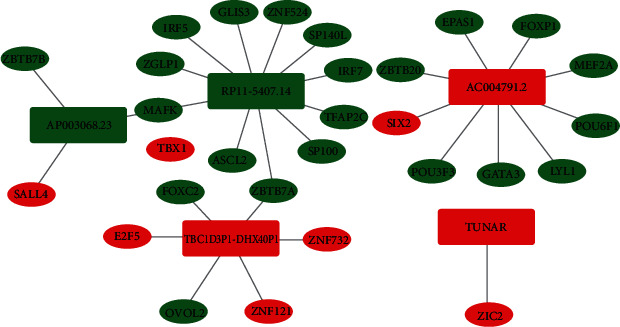
Regulatory network of transcription factors (TFs) and prognostic IrlncRNAs. The round rectangle represented lncRNAs and the ellipse represented TFs. The blue color indicated the downregulation of gene expression; the red color indicated the upregulation of gene expression.

To explore the potential regulatory mechanism of prognostic IrlncRNAs, co-expression analysis of differentially expressed TFs and prognostic IrlncRNAs was conducted. Thirty-one TFs-lncRNAs pairs were identified (correlation coefficient >0.4 and *p* < 0.01). Cytoscape software was used to visualize the TFs regulatory network (Figure 9). Among all TFs, both ZBTB7A and MAFK regulate two prognostic IrlncRNAs.

## 4. Discussion

RTK is a very rare kind of disease, which is little understood and poorly studied. At present, age and stage are the main prognostic factors for RTK [8, 9]. Several studies have reported that IrlncRNAs are not only predictors of tumor prognosis but also important targets for tumor treatment [31–34]. However, there is a lack of information about roles of IrlncRNAs in pRTK currently. According to the results of univariate and multivariate Cox analyses, this study established an IrlncRNA-based prognostic model for RTK. A risk score for each RTK patient was calculated based on the expression level of 5 IrlncRNAs, and according to the median value of this risk score, patients were divided into high-risk and low-risk groups. The overall survival time of the two-risk groups was significantly different. Univariate and multivariate Cox analyses of sex, stage, risk score, and age revealed that the risk score was an independent predictor of prognosis. ROC analysis demonstrated that the risk score was better than age, sex, and stage at predicting 1-year OS of RTK.

Our results suggest that 5 IrlncRNAs (AC004791.2, AP003068.23, RP11-54O7.14, TBC1D3P1-DHX40P1, and TUNAR) were significantly associated with the survival of pRTK patients. No studies have been reported concerning the relationships between any of these 5 lncRNAs and tumor prognosis through searching the PubMed database [35–37]. Exploring the regulatory mechanisms of prognosis-associated IrlncRNAs found that ZBTB7A and MAFK were important regulatory TFs. ZBTB7A is a member of the POK family of proteins that are known to function as transcriptional repressors of various different target genes [38]. Studies have reported that ZBTB7A plays both proto-oncogenic and tumor suppressive roles that depend on the cancer type and stage-specific situation and that targeting ZBTB7A could be a promising tumor growth inhibition approach [39–44]. MAFK (musculoaponeurotic fibrosarcoma oncogene family protein K) is a member of the small MAF family of transcription factors that form homodimers or heterodimers to regulate target gene expression [45]. Studies have shown that MAFK is closely associated with pancreatic cancer, acute myeloid leukemia, and osteosarcoma, implying it is a potentially new therapeutic targets for different cancers [46–48]. Both the five lncRNAs and the two TFs may be the potential targets for RTK to improve its prognosis.

The results of GSEA showed that promotion of tumor pathways, including cell cycle, DNA replication, pentose phosphate pathway, platinum drug resistance, and steroid biosynthesis, was significantly enriched in the high-risk group, but that antitumor pathway, including chemokine signaling pathway, cytokine-cytokine receptor interaction, natural killer cell-mediated cytotoxicity, Th1 and Th2 cell differentiation, and T cell receptor signaling pathway, was significantly enriched in the low-risk group [49–52]. These results further confirmed the credibility of our findings. Additionally, tumor microenvironment (TME) analysis showed that there was a difference in the characteristics of immune cell infiltration between the two groups, in that high-risk patients had higher proportions of macrophages, while low-risk patients had higher proportion of CD8_naive cells. NK cells, CD8 T cells, and gamma-delta T cells tended to be higher in low-risk group patients. In summary, our findings represent further steps in documenting the multiple functions of lncRNAs, which could affect the occurrence and development of tumors by influencing important physiological activities of tumor cells and tumor immune microenvironment.

Currently, effective chemotherapeutic drugs for RTK are limited, and the common drugs are mainly vinblastine and doxorubicin [9]. We used R package pRRophetic to evaluate the sensitivity (IC50) of high-risk and low-risk groups and found that there was no significant difference between them in this respect. We speculated that the reasons for this result may be two-fold. On the one hand, there were limited numbers of different chemotherapeutic drugs that could be analyzed in this database. Therefore, some effective drugs like cyclophosphamide, actinomycin D, ifosfamide, carboplatin, and etoposide are not included in the analysis. On the other hand, the number of patients was too small.

The main strength of our study is that for the first time, we found prognostic IrlncRNAs for pRTK. Our results could provide some tips to clinicians to evaluate the prognosis of each patient and make a reasonable treatment plan. Given our results suggesting two groups of patients with different TME, for patients without effective antitumor TME, we could consider employing immunotherapy to strengthen antitumor immunity to improve prognosis. In addition, our study yielded preliminary data on the mechanism of regulation and action of these prognosis-associated lncRNAs, which could provide avenues for basic research to explore the mechanism of tumor initiation to prevent and treat the disease. In summary, our study provides a theoretical basis for prevention, diagnosis, prognosis, and treatment of RTK. A limitation of our study is that in the small sample, we need a larger cohort to verify our findings. Also, this study has not been validated in the laboratory. In the future work, we plan to carry out in vitro experiments and conduct multicenter studies to verify the findings presented here.

## 5. Conclusion

Our prognostic model based on 5 IrlncRNAs can effectively predict the survival of RTK patients. In addition, the mechanism of regulation and action of prognosis-associated lncRNAs could provide avenues for basic research to explore the mechanism of tumor initiation and development to prevent and treat cancer.

## Figures and Tables

**Table 1:** Univariate Cox analysis of prognostic IrlncRNAs for overall survival.

Gene	HR (95% CI)	*p* value
AC004791.2	1.230 (1.106-1.368)	0.000137
AP003068.23	1.908 (1.293-2.817)	0.001144
RP11-54O7.14	1.033(1.014-1.053)	0.000661
RP11-680F8.1	1.492(1.140-1.952)	0.003540
TBC1D3P1-DHX40P1	2.442(1.374-4.340)	0.002351
TUNAR	1.097(1.044-1.152)	0.000242
XXbac-BPG308K3.5	1.334(1.082-1.644)	0.007042

Abbreviations: IrlncRNAs: immune-related long noncoding RNAs; HR: hazard ratio; CI: confidence interval.

**Table 2:** Multivariate Cox analysis of prognostic IrlncRNAs for overall survival.

Gene	Coefficient	HR (95% CI)	*p* value
AC004791.2	0.135096	1.145 (1.019-1.286)	0.023217
AP003068.23	0.487636	1.628 (1.052-2.521)	0.028711
RP11-54O7.14	0.035344	1.036 (1.012-1.061)	0.003086
TBC1D3P1-DHX40P1	1.108805	3.031 (1.612-5.696)	0.000573
TUNAR	0.063513	1.066 (0.994-1.143)	0.074167

Abbreviations: IrlncRNAs: immune-related long noncoding RNAs; HR: hazard ratio; CI: confidence interval.

## Data Availability

The entire sequencing profile data and the clinical data of pRTK patients in this study come from the Therapeutically Applicable Research to Generate Effective Treatments (TARGET, https://ocg.cancer.gov/programs/target) database, the immune gene list was downloaded from the Immunology Database and Analysis Portal (ImmPort, https://http://www.immport.org/), and a list of transcription factors (TF) was downloaded from the human transcription factors database (http://humantfs.ccbr.utoronto.ca/download.php).
